# Development of a Transcription Factor-Based Prognostic Model for Predicting the Immune Status and Outcome in Pancreatic Adenocarcinoma

**DOI:** 10.1155/2022/4946020

**Published:** 2022-05-05

**Authors:** Xianbin Zhang, Li Li, Peng Liu, Yu Tian, Peng Gong

**Affiliations:** ^1^Department of General Surgery & Institute of Precision Diagnosis and Treatment of Gastrointestinal Tumors, Shenzhen University General Hospital & Shenzhen University Clinical Medical Academy, Xueyuan Road 1098, 518055 Shenzhen, China; ^2^Guangdong Provincial Key Laboratory for Biomedical Measurements and Ultrasound Imaging, School of Biomedical Engineering, Shenzhen University Health Science Center, Xueyuan Road 1066, 518060 Shenzhen, China; ^3^Carson International Cancer Center & Guangdong Provincial Key Laboratory of Regional Immunity and Diseases, Shenzhen University Health Science Center, Xueyuan Road 1066, 518060 Shenzhen, China; ^4^Department of Epidemiology, Dalian Medical University, Lvshun Road 9, 116044 Dalian, China

## Abstract

Pancreatic adenocarcinoma (PAAD) is the most common primary malignancy of the pancreas. Growing studies indicate that transcription factors (TFs) are abnormally expressed in PAAD. We, therefore, aimed to evaluate the effect of TFs in PAAD and develop a TF-based prognostic signature for the patients. The expression of the TFs and the clinical characteristics were obtained from TCGA datasets. The levels of the TFs were evaluated in PAAD tissues or nontumor tissues. Kyoto Encyclopedia of Genes and Genomes (KEGG) was used to determine the potential function of the dysregulated TFs. To create a prognostic signature, we used univariate and multivariate Cox regression. In addition, the relationship between risk score and tumor microenvironment was analyzed. In this study, we observed 19 increased and 10 decreased TFs in PAAD tissues. KEGG assays indicated that dysregulated TFs were involved in transcriptional misregulation in cancer. Multivariate Cox analysis identified two prognostic factors, Zinc finger protein 488 and BCL11A; and we developed a risk score model by these two factors. The Kaplan-Meier estimator suggested that patients with high risk exhibited a shorter overall survival than those with low risk. The receiver operating characteristic curve proved that the accuracy of this prognostic signature was 0.686 in predicting the 5-year survival. In addition, we observed that the high score was distinctly related to advanced tumor stage and immune infiltrates. Taken together, we developed a novel TF-related model which could be applied as a potential prognostic tool for PAAD and may guide the choice of immunotherapies.

## 1. Introduction

Worldwide, more than half a million people die each year from pancreatic adenocarcinoma (PAAD), the most malignant tumor of the digestive system [1–3]. PAAD is associated with many risk factors including smoking, alcohol, gallstones, and chronic pancreatitis. Serum carbohydrate antigen is the most commonly used test to determine PAAD [4]. However, due to the low sensitivity and accuracy of this method, many patients are unable to be detected in the early stages of the tumor. This leads to the five-year overall survival no more than 10% [1, 4]. Therefore, it is important to develop novel tests for the early diagnosis of pancreatic cancer.

To activate or inhibit transcription of genes, transcription factors (TFs) attach to the transactivation or transrepression domains of DNA helix and regulate the genes' expression by turning on or turning off of the transcription [5–7]. Many biological processes, including the proliferation and death of cells, are regulated by transcription factors [8]. Notably, a variety of TFs was found to be dysregulated in many tumors such as cholangiocarcinoma [9], colon adenocarcinoma [10], and glioblastoma [11]. In addition, recent studies prove that the dysregulated expression of TFs may be involved in the immune functions of several types of carcinomas [12, 13]. This suggests that TFs might be promising diagnostic biomarkers and therapeutic targets in malignancies.

In this study, we aimed to investigate the expression of TFs in PAAD and develop a risk score model, which could be used to diagnose the PAAD and predict the prognosis of patients. Additionally, we evaluated if and how these TFs regulate immune infiltration. In conclusion, we developed a novel TF-based risk score model and this model will support the clinicians to choose the individualized therapeutic strategies for PAAD patients.

## 2. Materials and Methods

### 2.1. Biological Microarray Data

The expression of transcription factors was obtained from The Cancer Genome Atlas (TCGA) database. In this study, we choose the data of HTseq-FPKM, and the genetic expressions were presented as log_2_ (FPKM + 1). The patients whose follow-up period was less than 30 days were excluded from the study. Finally, we collected 1639 TFs from the study of Lambert et al. [14] and the survival analysis was performed in 172 PAAD patients.

### 2.2. Evaluating the Prognostic Value of TFs

To determine TFs that were differently expressed between PAAD and nontumor samples, the limma package was used. TFs with a log_2_ fold change (FC) > 1 and adjusted *P* values lower than 0.05 were identified as being differentially expressed. The false discovery rate was controlled by using the Benjamini-Hochberg method, and the R package “ggplot2” was used to construct the volcano charts [15]. The hierarchical cluster analysis was performed with the support of the heat map. The prognostic value of TFs was determined by the univariate and multivariate Cox regression analysis. All TFs were included in the univariate Cox regression, and the TFs which could significantly influence the prognoses of the patients were included in the multivariate Cox analysis.

### 2.3. Development and Validation of the TF-Related Prognostic Model

To develop the prognostic model, the independent prognostic TFs of PAAD patients were included and the model was developed with the support of the coefficient of the multiple Cox regression. To evaluate the prognosis of this model, the patients were divided into low- and high-risk categories. Kaplan-Meier curves and multivariate Cox regression were applied. The receiver operating characteristic curve (ROC) was used to determine the accuracy of this model in predicting the 5-year survival.

### 2.4. Evaluating the Relationship between TFs, Immune Infiltration, and Stroma

The infiltrations of immune cells were quantified by ssGSEA and immune scores [16]. The “GSEABase” and “GSVA” packages were used [17], and the enrichment score was obtained. The statistical differences between different groups were determined by Kruskal-Wallis tests. To evaluate the relationship between TFs and immune infiltration or stroma, two-way ANOVA was used.

### 2.5. Evaluating the Relationship between TFs and Stemness of Cells

Previous studies suggest that the stemness of carcinoma cells can be determined by the RNA-based stemness scores (RNAss) or the DNA methylation-based stemness scores (DNAss) [18]. We obtained the data from TCGA database and evaluated the stemness of the cancer cells by these scores. The values of the score were between 0 and 1. If the score is zero, this suggests that the cancer cells are well differentiated; if the score is one, this suggests that the cells are poorly differentiated and have strong stemness.

### 2.6. Statistical Methods

The R (version 4.0.3) was used to conduct all statistical analyses. The Wilcoxon tests were used to detect the statistically different expressions of TFs in PAAD tissues and nontumor tissues. The immune scores of different groups were determined by the Mann–Whitney *U* test, and the *P* values were adjusted by the Benjamini-Hochberg method. To determine the survival time of patients, the Kaplan-Meier curve and log-rank test were used. The univariate and multivariate Cox regressions were applied to identify the independent prognostic factor, and a TF-based prognostic score was developed by the coefficient of multivariate Cox regression. The statistical differences between scores of each group were determined by the Mann–Whitney *U* test. Differences with *P* ≤ 0.05 were considered to be significant.

## 3. Results

### 3.1. Identification of Differentially Expressed TFs in PAAD

**Figure 1:**
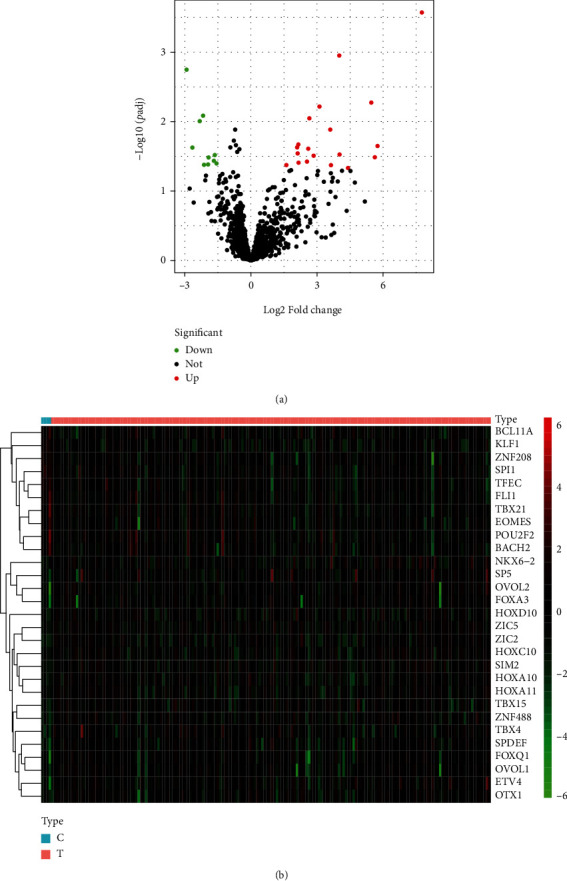
Identification of the dysregulated TFs in PAAD patients. (a) Volcano plot of upregulated and downregulated TFs. (b) The dysregulated TFs are showed in the heat map.

**Figure 2:**
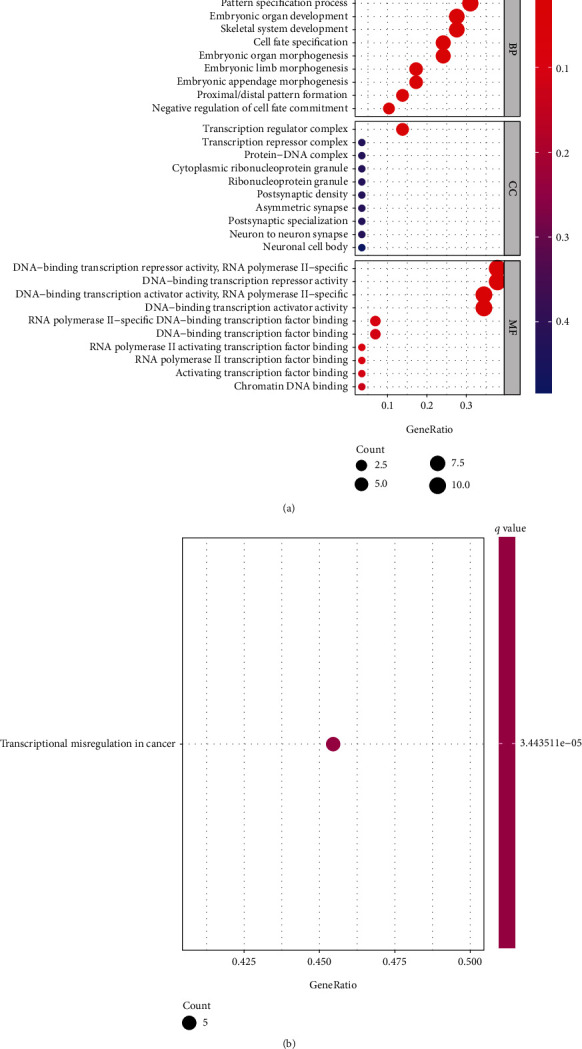
Functional analysis based on the dysregulated TFs between the two risk groups in PAAD. (a) Bubble graph for GO enrichment. (b) Bar plot graph for KEGG pathways.

The limma R package was used to determine TFs that exhibited a dysregulated level among 1639 profiles obtained from TCGA [19]. We observed that the expression of 19 TFs increased in the PAAD tissues and 10 TFs decreased (Figures 1(a) and 1(b)). These differentially expressed TFs were further studied by Gene Ontology (GO) and KEGG enrichment analysis. We observed that differentially expressed TFs were enriched in cell fate commitment, pattern specification process, transcription regulator complex, embryonic organ development, transcription repressor complex, protein-DNA complex, and DNA-binding transcription repressor activity (Figure 2(a)). KEGG assays indicated that the dysregulated TFs were involved in transcriptional misregulation in cancer (Figure 2(b)).

### 3.2. ZNF488 and BCL11A Are Independent Prognostic Factors of PAAD

**Figure 3:**
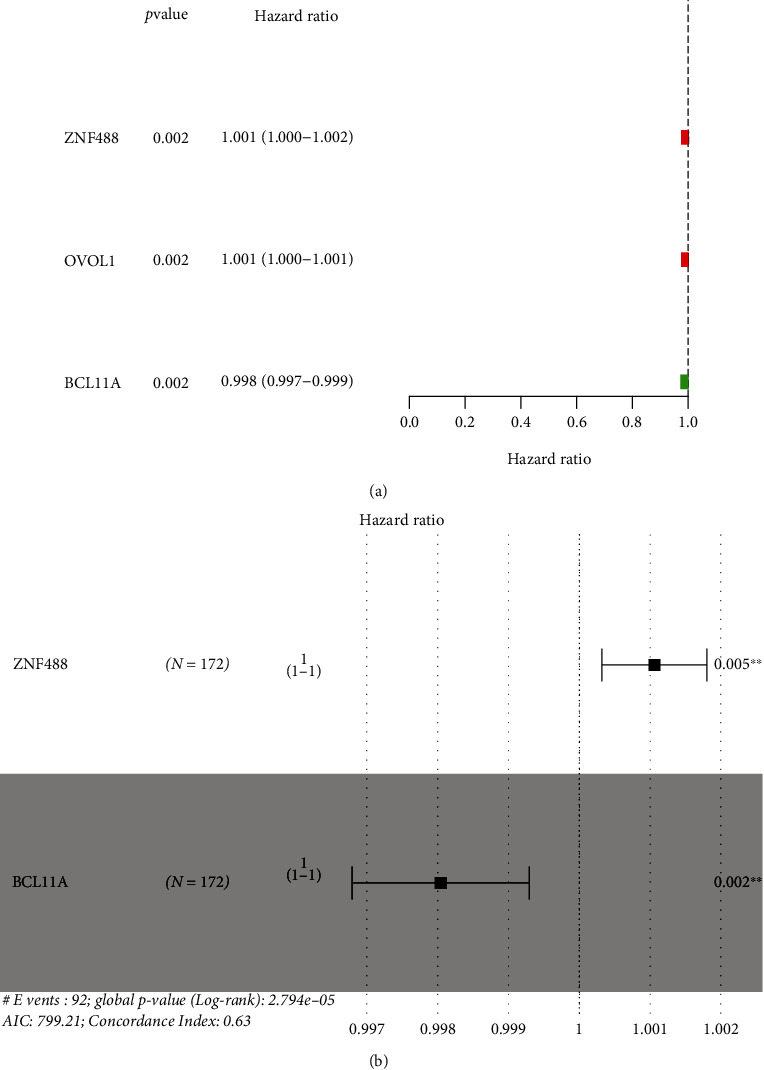
The survival-related TFs in PAAD patients. Forest plot of prognostic TFs by the use of (a) univariate assay and (b) multivariate assay.

To determine the prognostic TFs in PAAD, we performed the univariate Cox regression with the 29 dysregulated TFs in PAAD. We observed that Zinc finger protein 488 (ZNF488) and Ovo-like transcriptional repressor 1 (OVOL1) slightly increased the risk of death and BAF chromatin remodeling complex subunit, BCL11A, minor improved the prognosis of the patients (Figure 3(a)). The multivariate Cox regression suggested that ZNF488 and BCL11A were the independent prognostic factors for PAAD patients (Figure 3(b)).

### 3.3. Develop and Evaluate the TF-Based Prognostic Model

**Figure 4:**
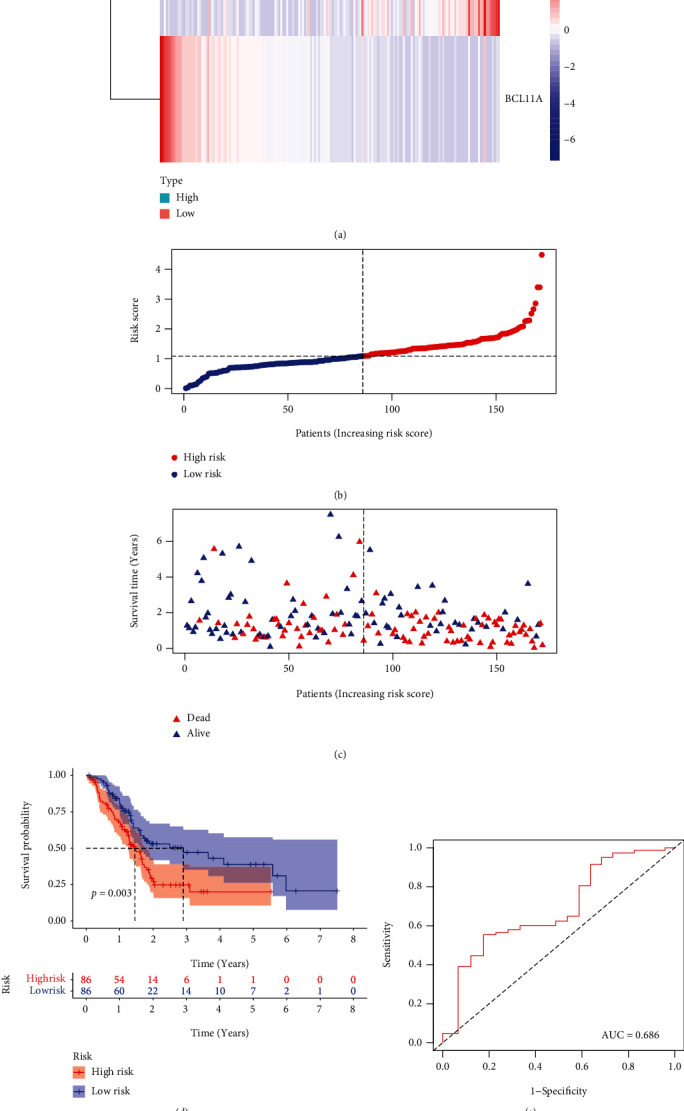
Development of the TF-based prognostic signature in PAAD. (a–c) Distributions of four-gene expression profiles of each patient, risk scores, and survival statuses of patients in the low-risk and high-risk groups. (d) Kaplan-Meier assays of the TF-related signature displaying worse survivals in the high-risk group. (e) ROC curve is applied to determine the diagnostic value of our model.

**Figure 5:**
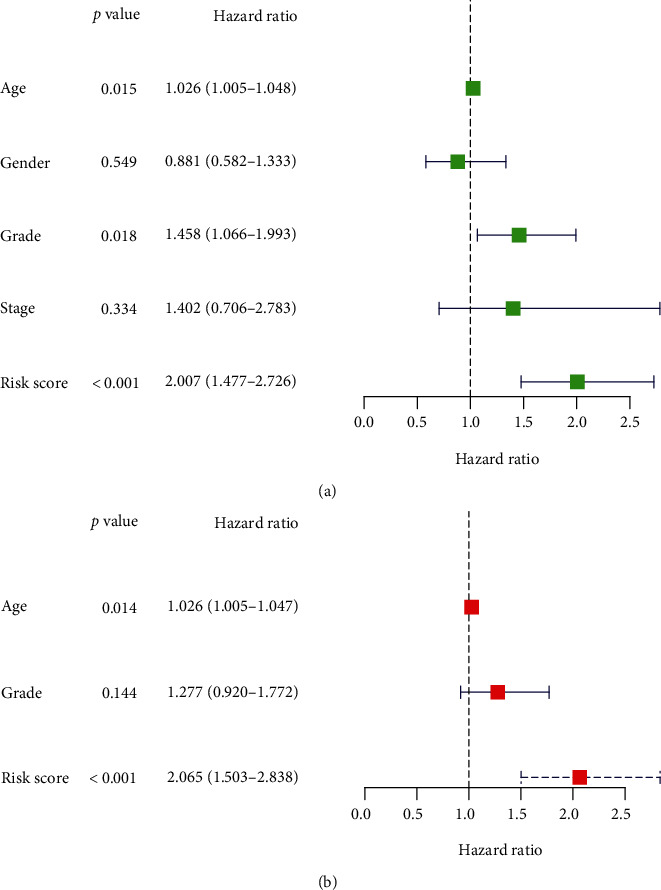
(a) Univariate and (b) multivariate assays of the relevancy between several clinical elements and overall survival of PAAD patients.

We used the coefficient of TFs and developed a model to predict the prognosis of patients (Score = 0.0010599 × levels of ZNF488 − 0.0019598 × levels of BCL11A). Based on the median score, all cases were divided into a high-risk group or a low-risk group. The expression of ZNF488 and BCL11A is presented in Figure 4(a), and the survival times of patients are presented in Figures 4(b) and 4(c). The Kaplan-Meier curve suggested that the survival time of patients with high-risk scores was significantly shorter than those with low-risk scores (Figure 4(d)). The ROC curve and the area under the curve indicated that the accuracy of this score in predicting the 5-year survival of patients was 0.686 (Figure 4(e)). In addition, the univariate and multivariate Cox regression suggested that the prognostic model was an independent prognostic factor of PAAD patients (Figures 5(a) and 5(b)).

### 3.4. The Relationship between the Prognostic Model and Clinical Features

**Figure 6:**
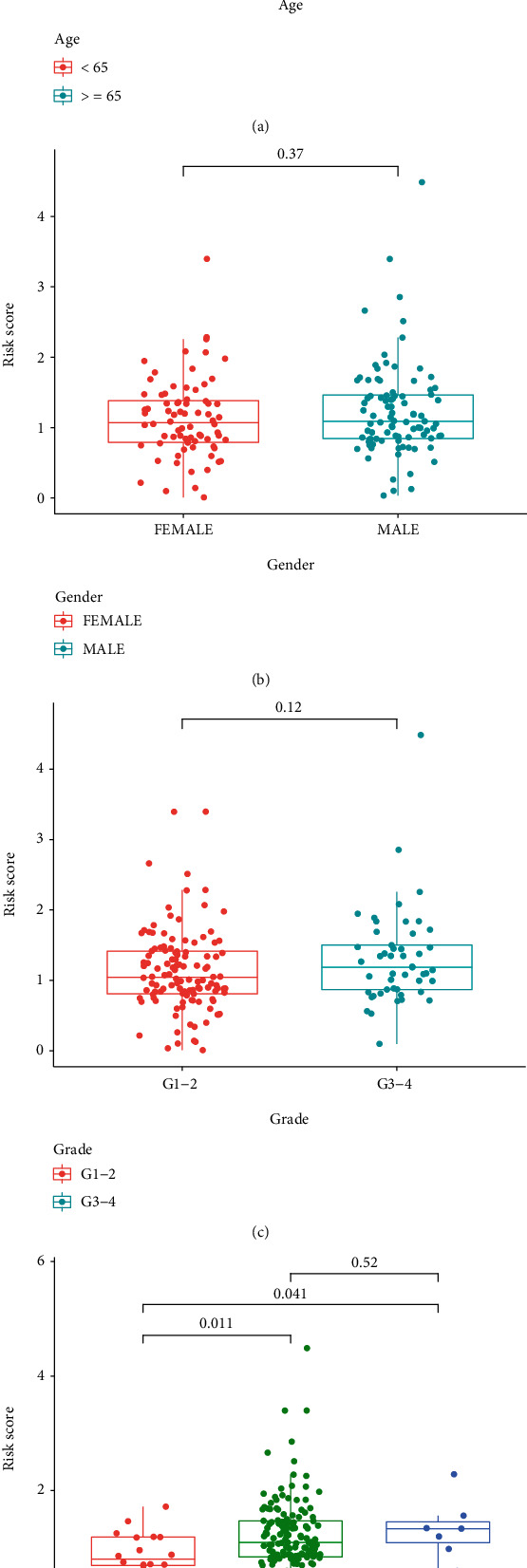
The association between risk score and (a) age, (b) gender, (c) grade, and (d) stage.

We further analyzed the association between the prognostic model and the clinical features. We observed that there were no significant differences between young patients and elderly patients (Figure 6(a)), female and male (Figure 6(b)), grade 1/grade 2 tumors, and grade 3/grade 4 tumors (Figure 6(c)). Interestingly, we observed that the risk of patients with the middle stage of tumors or advanced stage of tumors was significantly high than that with early stage of tumors (Figure 6(d)).

### 3.5. The Relationship between the Prognostic Model and Immune Infiltration

**Figure 7:**
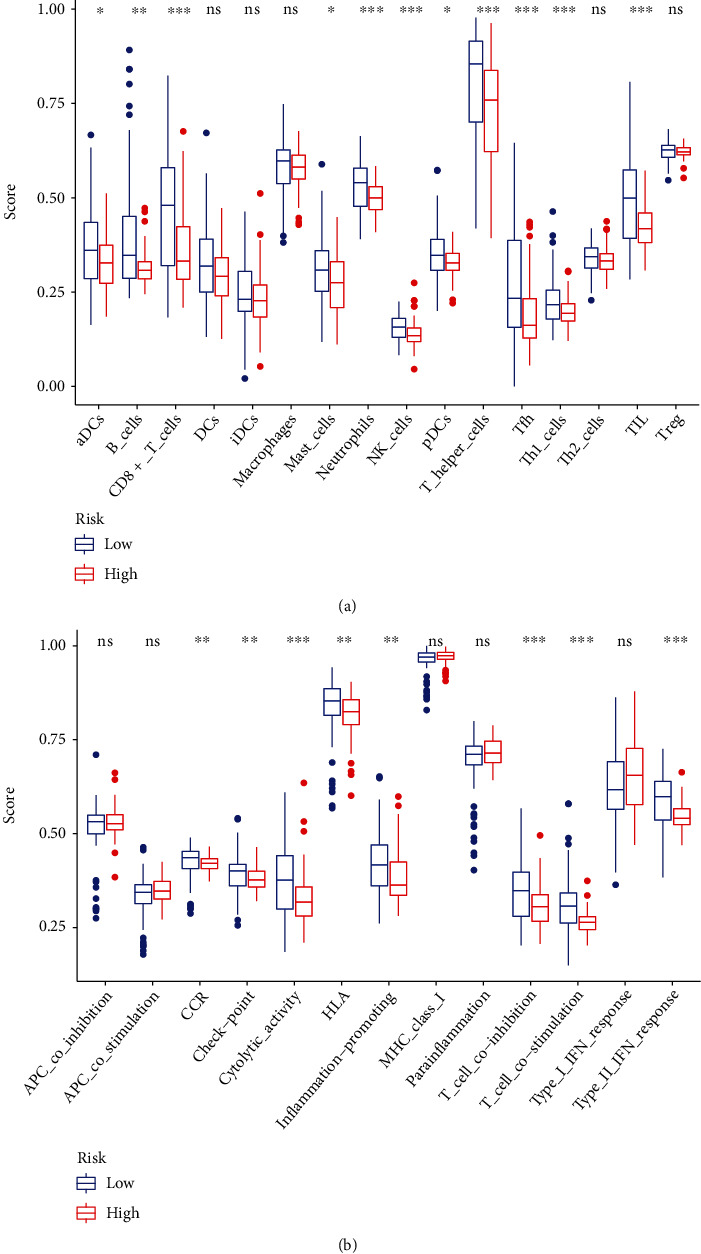
The relationships between risk score and tumor microenvironment. (a) The scores of 16 immune cells. (b) Boxplots were used to illustrate 13 immune-related processes.

**Figure 8:**
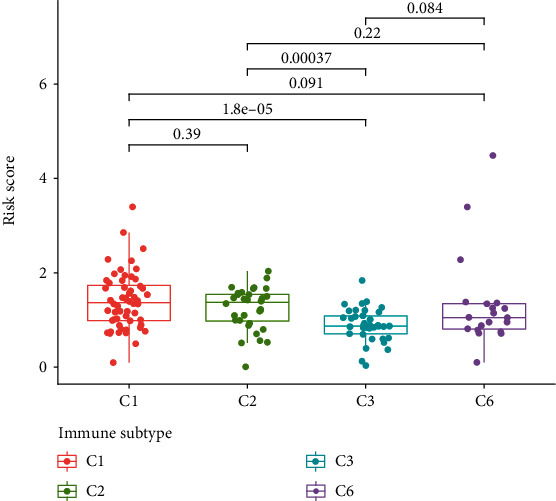
A high risk score was found to be related to C1, whereas a low risk score was related to C3 in our study of immune infiltration of PAAD in PAAD samples.

To evaluate if TFs were involved in the immune infiltration, we evaluated the relationship between the risk score and the immune status; we observed that compared to the low-risk group (blue box), the level of tumor-infiltrating lymphocytes (TIL), Th1 cells, T follicular helper (Tfh) cells, T helper (Th) cells, plasmacytoid dendritic (pDC) cells, natural killer (NK) cells, neutrophils, mast cells, CD8^+^ T cells, and B cells was significantly decreased in the high-risk group (red box, Figure 7(a)). Additionally, we observed that the level of type II IFN (IFN-*γ*), T cell costimulation, T cell coinhibition, inflammation-promoting, human leukocyte antigen (HLA), cytolytic activity, and checkpoint and CC chemokine receptor (CCR) were also significantly decreased in the high-risk group (red box, Figure 7(b)). To investigate the relationship between the risk score and the immune infiltration, the level of the risk score was evaluated in six types of immune infiltrations, C1 (wound healing), C2 (INF-*γ* dominant), C3 (inflammatory), C4 (lymphocyte depleted), C5 (immunologically quiet), and C6 (TGF-*β* dominant). We observed that C3 had a low risk score when compared to C1 or C2 (Figure 8).

### 3.6. The Relationship between the Prognostic Model and Stemness, Immunological, or Stromal Microenvironment

**Figure 9:**
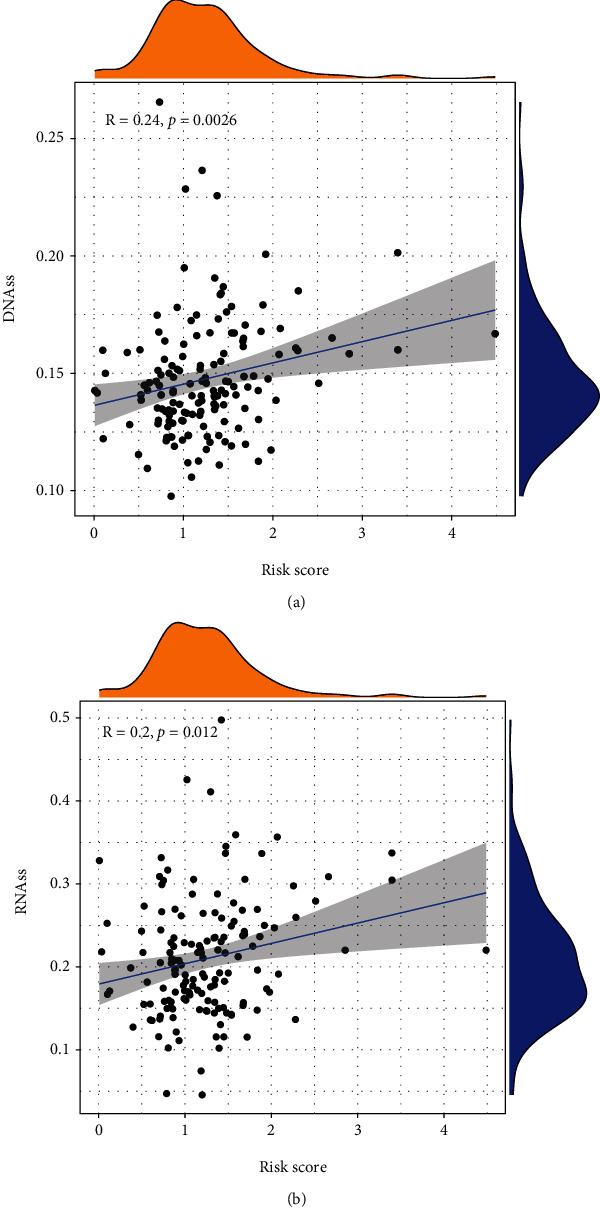
The relationships between risk score and (a) DNAss and (b) RNAss.

**Figure 10:**
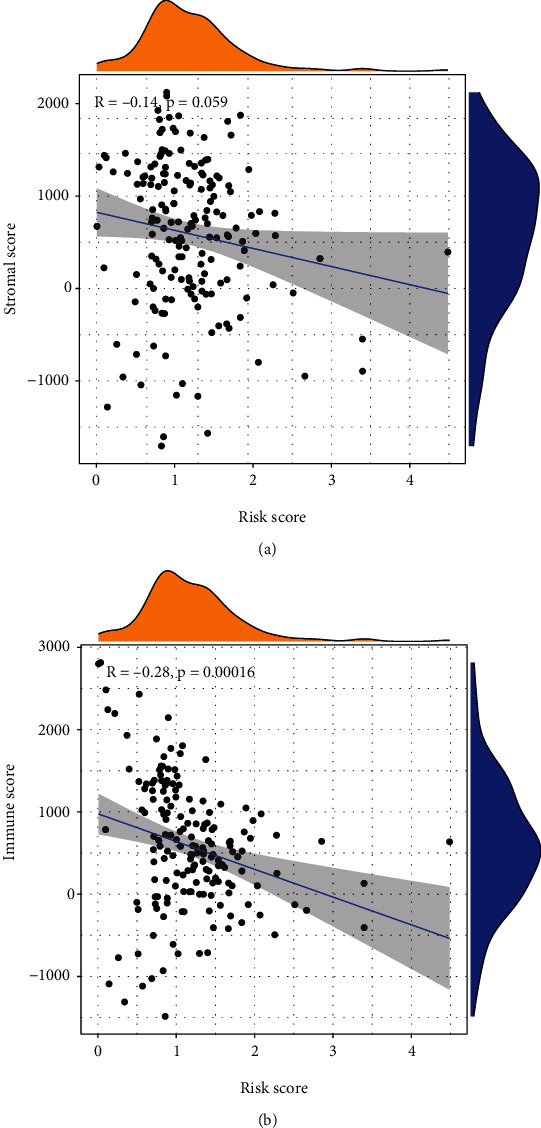
The relationships between risk score and (a) stromal score and (b) immune score.

To evaluate the relationship between the risk score and stemness, immunological, or stromal microenvironment, we calculated the Spearman rank correlation coefficient of the risk score and the scores of stemness, immunology, or stroma. We observed that the risk score was positively associated with RNA-based stemness scores (RNAss, Figure 9(a)) or the DNA methylation-based stemness scores (DNAss, Figure 9(b)) and negatively associated with the stromal score (Figure 10(a)) and immune score (Figure 10(b)). This suggested that the risk score increased the stemness of tumors and impaired the immunological or stromal microenvironment.

## 4. Discussion

PAAD is the most aggressive and fatal tumor [20, 21]. Due to the lack of a sensitive and specific test of PAAD, the tumors have already spread from the pancreas to the liver and lung [22]. Thus, it needs to develop a novel test that can diagnose PAAD at an early stage and monitor the treatment response. Previous studies reported that TFs are involved in the genesis, development, and metastasis of several tumors, [23, 24] and therefore, TFs are promising biomarkers for the diagnosis of PAAD [25].

In the present study, we observed that ZNF488 and BCL11A were independent prognostic variables of PAAD patients and we developed a predictive model by using these two TFs. The predictive model proved that patients with high-risk scores had a short overall survival. Additionally, the ROC curve indicated that this risk score had an acceptable accuracy in predicting the 5-year survival. The univariate and multivariate Cox regression confirmed that ZNF488 and BCL11A were independent prognostic factors for PAAD patients. This was supported by previous studies [26, 27]. For example, Qiu et al. proved that ZNF488 promotes the invasion and migration of PAAD cells by activating the Akt/mTOR signaling pathway [26]. Zhou et al. found that overexpression of BCL11A promoted the growth of laryngeal squamous cell carcinoma [27]. In addition, we evaluated if and how ZNF488 or BCL11A was involved in the immunological microenvironment. We observed that the level of CD8^+^ T cells significantly decreased in the high-risk group. It is well known that CD8^+^ T cells are the cytotoxic T lymphocytes that kill the carcinoma cells [28]. This may be a possible mechanism that the patients in the high-risk group have a poor prognosis.

It is reported that cancer stem cell-like cells are master contributors to the poor survival of PAAD [29]. We, therefore, evaluated the relationship between the risk score and the stemness of cancer cells. In the process of tumor growth, some tumor cells lose their differentiation potential and gradually have the characteristics of progenitor cells and stem cells. This is due to the high levels of methylation in the DNA of some genes, and previous studies suggest that RNAss and DNAss can accurately reflect the stemness of tumor cells [18]. We observed that the TF-based score was positively correlated with the RNAss or DNAss. This indicates that ZNF488 or BCL11A increases the stemness of cancer cells. To our knowledge, no study reports how ZNF488 regulates the stemness of PAAD cells. Zong et al. find that ZNF488 is an independent prognostic factor of nasopharyngeal carcinoma, and it promotes the adhesion and proliferation of cells by the IV/FAK/AKT/Cyclin D1 pathway [30]. In the future, additional studies could evaluate if IV/FAK/AKT/Cyclin D1 is involved in the ZNF488-induced stemness. Zhu et al. report that BCL11A could enhance stemness by activating the Wnt/*β*-catenin signaling [31]. Thus, the combinational therapeutic strategies that target the BCL11A and Wnt/*β*-catenin signaling pathway are a promising treatment for PAAD patients.

## 5. Conclusion

In conclusion, based on ZNF488 and BCL11A, we developed a prognostic model and the accuracy of this model was 0.686 in predicting the 5-year survival. In addition, we observed that ZNF488 and BCL11A were positively related to the advanced tumor stage and stemness. Targeting ZNF488 and BCL11A may be a promising strategy for the treatment of PAAD.

## Data Availability

The data used to support the findings of this study are available from the corresponding author upon request.
